# Functional Analysis of a CTL-X-Type Lectin CTL16 in Development and Innate Immunity of *Tribolium castaneum*

**DOI:** 10.3390/ijms241310700

**Published:** 2023-06-27

**Authors:** Jingxiu Bi, Yutao Wang, Rui Gao, Pingxiang Liu, Yuying Jiang, Lei Gao, Bin Li, Qisheng Song, Mingxiao Ning

**Affiliations:** 1 Laboratory of Quality and Safety Risk Assessment for Agro-Products of the Ministry of Agriculture (Jinan), Institute of Quality Standard and Testing Technology for Agro-Products, Shandong Academy of Agricultural Sciences, Jinan 250100, China; jxbi428@163.com (J.B.); zhbswangyutao@shandong.cn (Y.W.); 15665827772@163.com (R.G.); liupingxiangtri@163.com (P.L.); gaolei@sdsnykxy.wecom.work (L.G.); 2 Jiangsu Key Laboratory for Biodiversity and Biotechnology, College of Life Sciences, Nanjing Normal University, Nanjing 210023, China; libin@njnu.edu.cn; 3 Division of Plant Science and Technology, University of Missouri, Columbia, MO 65211, USA; songq@missouri.edu

**Keywords:** *Tribolium castaneum*, C-type lectin, bacterial agglutination, antimicrobial peptides

## Abstract

C-type lectins (CTLs) are a class of proteins containing carbohydrate recognition domains (CRDs), which are characteristic modules that recognize various glycoconjugates and function primarily in immunity. CTLs have been reported to affect growth and development and positively regulate innate immunity in *Tribolium castaneum*. However, the regulatory mechanisms of TcCTL16 proteins are still unclear. Here, spatiotemporal analyses displayed that *TcCTL16* was highly expressed in late pupae and early adults. *TcCTL16* RNA interference in early larvae shortened their body length and narrowed their body width, leading to the death of 98% of the larvae in the pupal stage. Further analysis found that the expression level of muscle-regulation-related genes, including *cut*, *vestigial*, *erect wing*, *apterous*, and *spalt major*, and muscle-composition-related genes, including *Myosin heavy chain* and *Myosin light chain*, were obviously down-regulated after *TcCTL16* silencing in *T. castaneum*. In addition, the transcription of *TcCTL16* was mainly distributed in the hemolymph. *TcCTL16* was significantly upregulated after challenges with lipopolysaccharides, peptidoglycans, *Escherichia coli*, and *Staphylococcus aureus*. Recombinant CRDs of TcCTL16 bind directly to the tested bacteria (except *Bacillus subtilis*); they also induce extensive bacterial agglutination in the presence of Ca^2+^. On the contrary, after *TcCTL16* silencing in the late larval stage, *T. castaneum* were able to develop normally. Moreover, the transcript levels of seven antimicrobial peptide genes (*attacin2*, *defensins1*, *defensins2*, *coleoptericin1*, *coleoptericin2*, *cecropins2*, and *cecropins3*) and one transcription factor gene (*relish*) were significantly increased under *E. coli* challenge and led to an increased survival rate of *T. castaneum* when infected with *S. aureus* or *E. coli*, suggesting that TcCTL16 deficiency could be compensated for by increasing AMP expression via the IMD pathways in *T. castaneum*. In conclusion, this study found that TcCTL16 could be involved in developmental regulation in early larvae and compensate for the loss of CTL function by regulating the expression of AMPs in late larvae, thus laying a solid foundation for further studies on *T. castaneum* CTLs.

## 1. Introduction

Insect innate immune systems utilize soluble and membrane-bound receptors to recognize pathogen-associated molecular patterns (PAMPs) on the surface of pathogenic microorganisms [1]. Beta-1, 3-glucan recognition proteins (βGRPs), peptidoglycan-recognition proteins (PGRPs), Gram-negative binding proteins (GNBPs), and lectins bind to glycolipids, polysaccharides, and glycoproteins on pathogen surfaces to induce a cascade of downstream defense responses [2,3]. Based on their domain action mechanisms and architectures, animal lectins are classified into C-type lectins (CTLs), β-galactose-specific lectin, immunoglobulin-type lectin, mannose-6-phosphate receptors, pentraxins, etc. [4]. CTLs are one of the largest and most diverse families of lectins in vertebrates and invertebrates. They are characterized by the requirement of Ca^2+^ to maintain carbohydrate-binding structures and activities. Each CTL has one or more carbohydrate recognition domains (CRD), known as CTL domains, which are composed of β-sheets, α-helices, and loops [5]. 

Animal CTLs are usually involved in innate immunity, including regulating anti-fungal immunity [6], enhancing melanization, phagocytosis, and encapsulation [7,8,9], prophenoloxidase activation [10], bacterial clearance [8], and regulation of the production of antimicrobial peptides (AMPs) [11]. Based on the number of CRDs and domain architectures, insect CTLs are classified into three groups: the CTL-S type, the immulectin type, and the CTL-X type [12]. Normally, CTL-S has a single CRD, while immulectin has at least two CRD domains. Previous studies found that *Drosophila melanogaster* lectin 2 (DL2) and DL3, belonging to the CTL-S type, could agglutinate *E. coli* and enhance melanization and encapsulation in vitro [13,14]. Regenectin (CTL-S) was involved in the regeneration of cockroach legs [15]. *Helicoverpa armigera* CTL-3 [16] and *Ostrinia furnacalis* IML-10 [17], belonging to the immulectin type, could promote cellular encapsulation and aggregation. Different from the above two groups, besides the CRD domain, the CTL-X group contains other functional domains, such as an extracellular domain (CUB), complement control protein (CCP) modules, immunoglobulin modules (Ig), a chitin-binding domain (CBM), the discoidin domain family (F5/8 type C domain), and an epidermal-growth-factor-like domain (EGF) [18]. Studies have found that CTL-X CTLs are mainly involved in insect growth and development. For instance, furrowed (a CTL-X type lectin) in *D. melanogaster* is required for the development of bristles and eyes [19,20]. Similarly, contactin (a CTL-X type lectin) is also required for paracellular barrier function and septate junction organization [21,22,23]. However, it is unknown whether CTL-X-type CTLs are also involved in the innate immunity and/or development of *Tribolium castaneum*. 

*T. castaneum*, a destructive insect pest of stored grain-based products, is a model organism widely used in immunological, developmental, and evolutionary biology research [24,25]. A total of 17 CTL genes have been identified in the *T. castaneum* genome using a bioinformatics approach [26]. Earlier studies have found that TcCTL3 plays a vital role in the immune response towards bacterial infection by influencing the expression of AMPs [27]. TcCTL5 was found to participate in the innate immunity and individual movement of *T. castaneum* [28]. In addition to its role in the immune response, TcCTL12 was found to have extensive functions in the regulation of development in *T. castaneum* [1]. There is no doubt that these CTLs can induce protective responses during bacterial infections in *T. castaneum*. In contrast, viruses are able to utilize mosquito CTLs to facilitate infection. A previous study identified an *Aedes aegypti* CTL, *mosGCTL*-*1*, that facilitated West Nile virus (WNV) infection in vitro and in vivo. The expression of *mosGCTL*-*1* was upregulated after WNV infection, and it interacted with *mosPTP*-*1*, which enabled viral attachment to cells and facilitated viral entry [29]. Moreover, *Aedes aegypti mosGCTL* genes were first identified by Liu et al. [30] as key susceptibility factors for dengue virus (DENV)-2 infection. Further study found that *mosGCTL*-*3* could significantly enhance viral infectivity by interacting with the DENV-2 envelope protein in vitro and in vivo. However, it is not clear whether there are genes in the CTL family of *T. castaneum* that negatively regulate the immune response during bacterial infection and what the underlying regulatory mechanism is.

In the past 2–3 decades, many studies on the functions of insect CTLs in innate immunity have been published. Nevertheless, the function of *TcCTL16* has not been studied yet. To explore the function of TcCTL16, a CTL-X type CTL, we cloned the full-length cDNA of *TcCTL16* from *T. castaneum* and investigated its developmental and tissue-specific expression profiles, as well as its response to different elicitors. Most importantly, we investigated its role in development and pattern recognition, agglutination, and AMP regulation after bacterial infection. This research lays a solid foundation for further studies of *T. castaneum* CTLs.

## 2. Results

### 2.1. Domain Organization, Structure, and Evolutionary Relationships among CTLs

**Figure 1:**
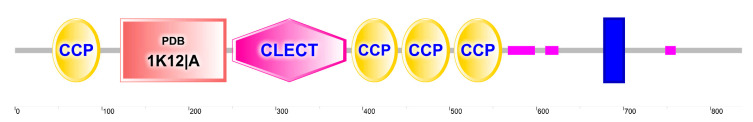
Schematic presentation of TcCTL16 protein showing all six domains. Four CCPs, one K12A domain, one CRD and one transmembrane helix region (blue box) were identified in TcCTL16 protein. The pink box is low complexity region.

The open reading frame (ORF) of *TcCTL16* is 2511 bp in length and encodes a protein of 836 amino acids (Figure S1), with a theoretical molecular mass of 92.71 kD and an isoelectric point of 8.28 (Gene ID:TC002984). Four CCPs (the first from C43 to C98, the second from C387 to C440, the third from C445 to C500, and the fourth from C505 to C560), one CRD (from P251 to Q381) and one transmembrane helix region (from I678 to V700) were identified in the protein (Figure 1). As shown in Figure S2, CTL-S5, 6, and 13 contain the QPD, WLD, and WHD motifs, respectively, which may bind a variety of carbohydrates. The carbohydrate-recognition motif in other CTLs presents a mutated signature, such as WSA, WNS, YFR, etc., and thus, their characteristics need further investigation (Figure S2).

Phylogenetic analysis showed that 17 CTLs of *T. castaneum* formed close lineal homology groups with CTLs in Hymenoptera (*H. laboriosa* and *A. glabripennis*), Lepidoptera (*M. sexta* and *B. mori)*, and Diptera (*A. gambiae* and *D. melanogaster*) (Figure S3). Our results showed that these genes belong to lineal homologues, closely clustered with genes of *Diptera*, *Hymenoptera,* and *Lepidoptera*. Some CTLs of *D. melanogaster*, *A. gambiae,* and *A. glabripennis* were found to be isolated into a single branch, suggesting the gene duplication of CTLs in the species, while there were no species-specific genes in *M. sexta*, *B. mori*, or *T. castaneum*. IML 3 and some CTL-S-type lectins (AgmCTL11, DmCTL29, BmCTLS6, MsCTLS5, HaKOC60049.1, AglXP0233132226.1, and TcCTL8) are clustered into one branch, suggesting that TcCTL3 may originate from CTL-S-type lectins (Figure S3).

### 2.2. Spatiotemporal Expression of TcCTL16 

**Figure 2:**
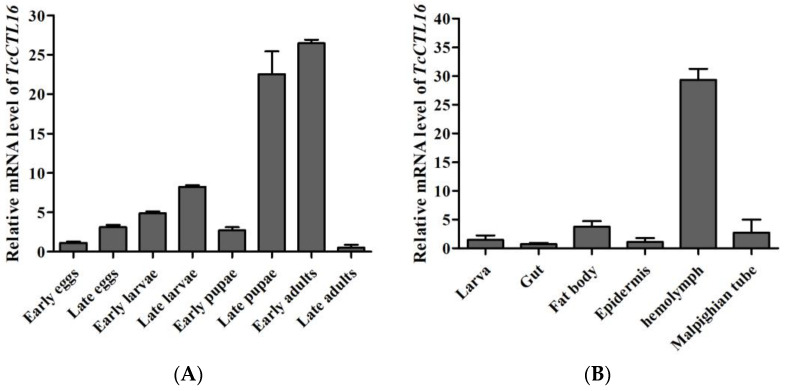
The spatiotemporal expression of *TcCTL16*. (**A**) Developmental expression patterns of *TcCTL16*. The RNA extract from whole body was used for the qRT-PCR at different developmental stages. The relative expressions of the target transcripts in the early egg served as the calibrator for the developmental expression profiling. (**B**) Tissue-specific expression patterns of *TcCTL16* in gut, fat body, epidermis, hemolymph, and Malpighian tubule from the 15-day-old larvae. The relative expression of the target transcripts in larva was employed as the calibrator for the tissue-specific expression profiling.

qRT-PCR analysis revealed that *TcCTL16* was transcribed throughout all developmental stages, showing a low level at the LA stage and high levels at the LP and EA stages (Figure 2A), suggesting *TcCTL16* could play an important role in the growth and development of *T. castanuem*. Moreover, the expression levels of *TcCTL16* varied in different larval tissues, with the highest expression in the hemolymph, while it exhibited low transcription levels in gut, fat body, epidermis, and Malpighian tube tissues (Figure 2B). 

### 2.3. TcCTL16 Silencing in Early Larvae Influence Growth and Development 

**Figure 3:**
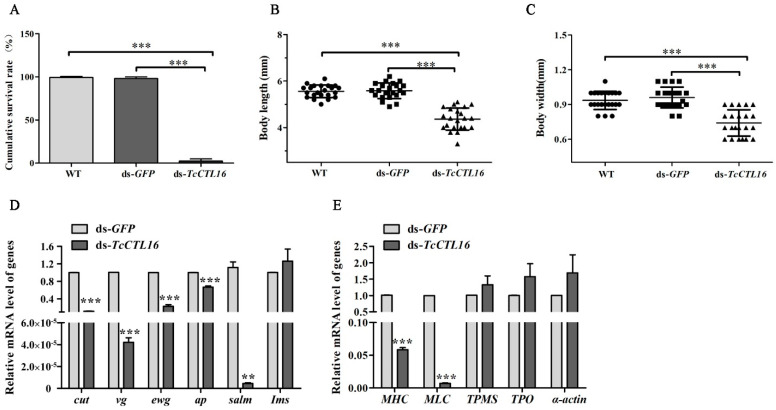
Statistics of survival rate (**A**), body length (**B**), and body width (**C**) compared with WT, ds-*GFP*, and ds-*TcCTL16* groups. The expression levels of genes involved in muscle regulation (**D**) and muscle composition (**E**) after *TcCTL16* RNAi. WT: wild type; ds-*GFP*: *GFP*-RNAi larvae; ds-*TcCTL16*: *TcCTL16*-RNAi larvae. *Tcrps3* was used as a reference gene. The results are the mean and standard errors of three biological replications (*n* = 3). Asterisk denotes significant differences (** *p* < 0.01; *** *p* < 0.001).

When we applied a knockdown of *TcCTL16* on early larvae (12-day-old larvae), the cumulative survival rate of *T. castaneum* was only 2% (Figure 3A). In addition, *TcCTL16* silencing slowed down the growth and development of the larvae, shortened their body length (Figure 3B), and narrowed their body width (Figure 3C); this all occurred when the 12-day-old larvae were injected with ds-*TcCTL16*. Next, the expression of genes associated with muscle regulation and composition was examined after *TcCTL16* silencing. Our results showed that the expression levels of muscle-regulation-related genes, including *cut*, *vestigial* (*vg*), *erect wing* (*ewg*), *apterous* (*ap*), and *spalt major* (*salm*), were obviously down-regulated compared with the control group (Figure 3D). Genes associated with muscle composition, including *Myosin heavy chain* (*Mhc*) and *Myosin light chain* (*Mlc*), were also decreased (Figure 3E). These results indicate that *TcCTL16* may influence the growth and development of *T. castaneum* by regulating genes associated with muscle regulation and composition. 

### 2.4. TcCTL16 Responses to Polysaccharide or Bacterial Challenge

**Figure 4:**
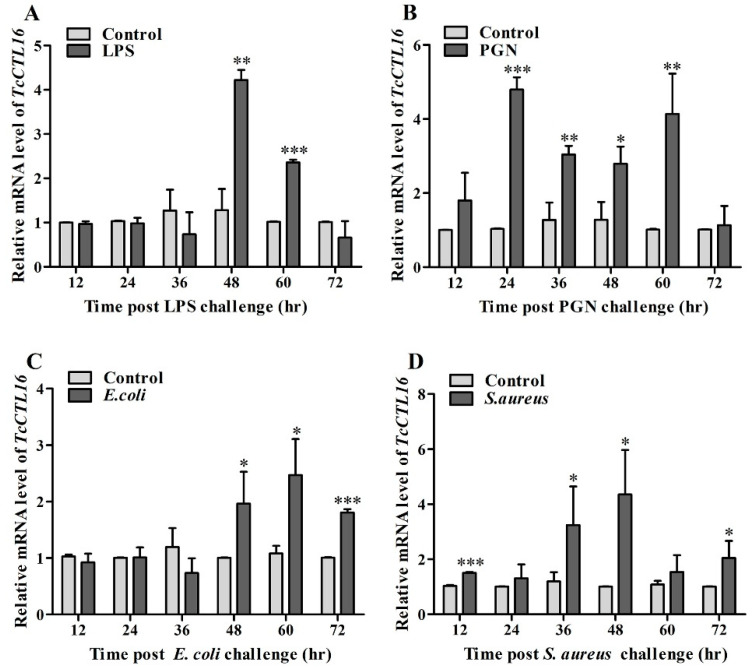
qRT-PCR analysis of *TcCTL16* expression 12 h to 72 h after LPS (**A**), PGN (**B**), *E. coli* (**C**) and *S. aureus* (**D**) challenge. Asterisks indicate significant differences (* *p* < 0.05, ** *p* < 0.01, *** *p* < 0.001) compared with values of the control. The results are the mean and standard errors of three biological replicates.

The mRNA levels of *TcCTL16* in the LPS-challenged group and PGN-challenged group were significantly upregulated from 48 to 60 h and 24 to 60 h, respectively (Figure 4A,B). In addition, for the *E. coil*-challenged group, the transcript level of *TcCTL16* increased significantly from 48 to 72 h (Figure 4C). The transcript level of *TcCTL16* increased significantly at 12, 36, 48, and 72 h after the *S. aureus* challenge (Figure 4D). These results illustrated that *TcCTL16* could take part in the innate immune responses of *T. castanuem*.

### 2.5. Production of rTcCTL16 CRD and Its Binding and Agglutination Capacity to Microbes

**Figure 5:**
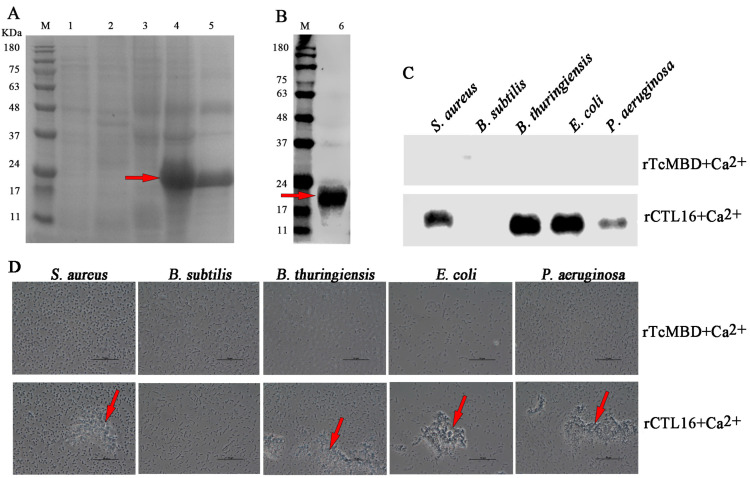
SDS-PAGE (**A**) and Western blotting (**B**) analysis of the rCTL16 protein. Lane M: the protein molecular weight marker; lane 1: the supernatant protein of *E. coli* with pET-28a; lane 2: precipitated protein from *E. coli* with pET-28a; lane 3: the supernatant protein of *E. coli* with pET-28a-TcCTL16; lane 4: the precipitated protein from *E. coli* with pET-28a-TcCTL16; lane 5: purified recombinant TcCTL16 protein; lane 6: Western blot based on the sample from lane 5. (**C**) Binding of each recombinant protein rCTL16 to microorganisms (*S. aureus*, *B. subtilis*, *B. thuringiensis*, *E. coli*, and *P. aeruginosa*) were detected by Western blot. (**D**) Microorganism agglutination activities of rCTL16 with either the absence or presence of Ca^2+^. Three Gram-positive (*S. aureus*, *B. subtilis*, and *B. thuringiensis*) and two Gram-negative bacteria (*E. coli* and *P. aeruginosa*) are shown. rTcMBD was used as a negative control protein. Agglutination reactions were observed using a compound light microscope with a 1000× objective. The red arrows indicate the agglutination region.

rCTL16 was expressed in inclusion bodies from *E. coli* BL21 (DE3) (Figure 5A, lane 4) and purified through a Ni Sepharose 6 Fast Flow column (Figure 5A, lane 5). Western blot assay verified the successful expression of rCTL16 (Figure 5B, lane 6). To investigate whether rCTL16 could bind to Gram^+^ or Gram^−^ bacteria, a bacterial binding assay was performed. rCTL16 exhibited strong binding activities toward Gram^+^ bacteria (*S. aureus* and *B. thuringiensis*) and Gram^−^ bacteria (*E. coli* and *P. aeruginosa*) but had no binding ability to *B. subtilis* (Figure 5C). Moreover, as shown in Figure 5D, rCTL16 protein could agglutinate Gram^+^ bacteria (*S. aureus* and *B. thuringiensis*) and Gram^−^ bacteria (*E. coli* and *P. aeruginosa*) but had no agglutinating ability to *B. subtilis*.

### 2.6. TcCTL16 Silencing on Late Larvae Does Not Affect Viability of Beetles

**Figure 6:**
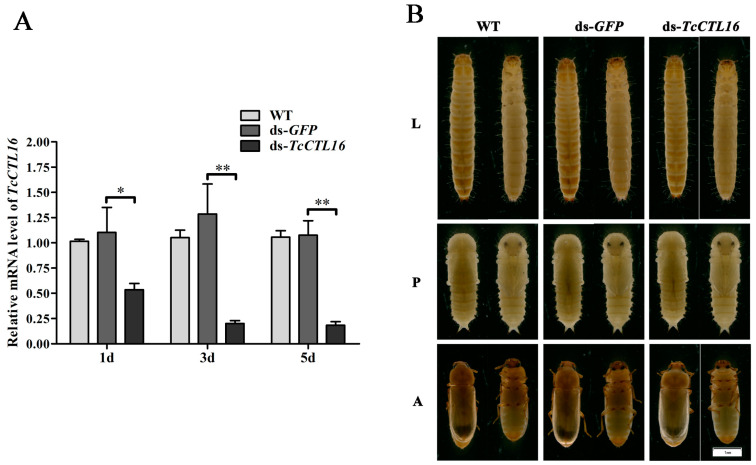
TcCTL16 expression patterns on 1st, 3rd, and 5th days in the dsRNA-TcCTL16-treated insects. Asterisks indicate significant differences (* *p* < 0.05, ** *p* < 0.01) compared with values of the ds-*GFP*. (**A**). Phenotypes of WT, ds-GFP, and ds-TcCTL16 groups. L, late larvae; P, late pupae; A, early adults. Pictures were taken by Olympus DP-72 digital camera (Olympus Corporation, Tokyo, Japan) through an Olympus SZX-16 microscope (Olympus Corporation). The larvae were photographed at ×16 magnification, while the pupae and adults were at ×20 magnification (**B**).

The efficiency of *TcCTL16* RNAi reached 65% on the first day and 80% on the third and remained at 80% on the fifth day (Figure 6A) on late larvae (15-day-old larvae). Interestingly, no significant impact on the 15-day-old larvae viability of ds-*TcCTL16*- and ds-*GFP*-injected beetles was observed (Figure 6B). 

### 2.7. Effects of TcCTL16 Gene Knockdown on Defense against Bacterial Infection 

**Figure 7:**
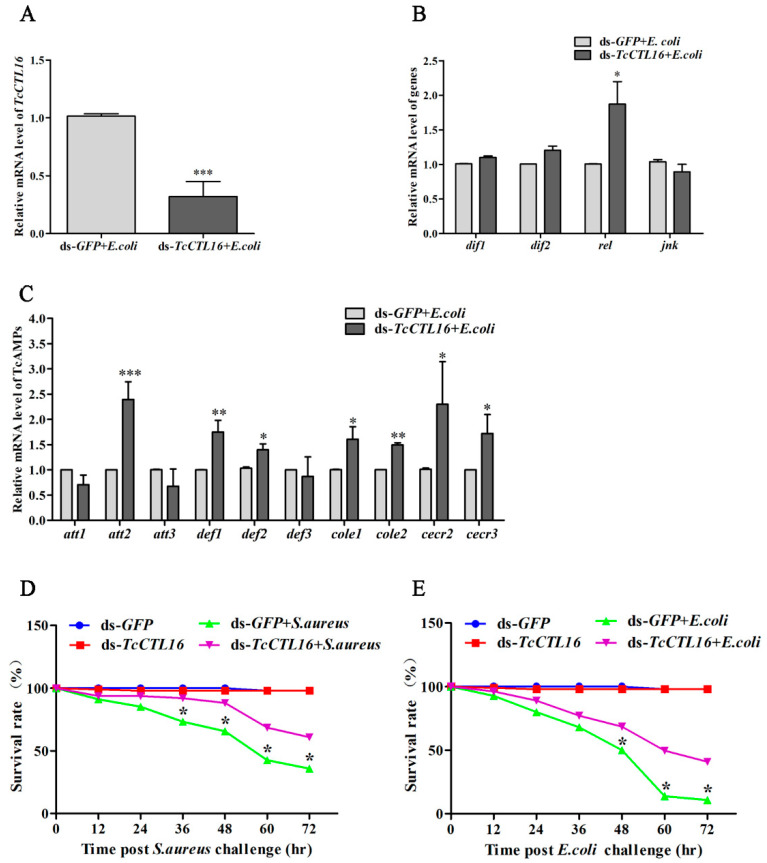
TcCTL16 expression patterns in response to *E. coli* challenge in the dsRNA-TcCTL16-treated insects (**A**). The expression patterns of transcription factor (**B**) and AMP (**C**) genes upon *E. coli* challenge after successful RNAi of *TcCTL16*. The survival rate of *TcCTL16*-RNAi larvae infected with *S. aureus* (**D**) and *E. coli* (**E**). The number of dead beetles was recorded every 12 hr post-challenge. The control group was ds-*GFP* + bacterium (*S. aureus* or *E. coli*). Statistical comparisons were performed between the ds-*GFP*-injected and ds-*TcCTL16*-injected insects. *Tcrps3* was used as a reference gene. Asterisks indicate significant differences (* *p* < 0.05, ** *p* < 0.01, *** *p* < 0.001) compared with values of the control. The results are the mean and standard errors of three biological replicates.

As shown in Figure 7A, silencing *TcCTL16* significantly inhibited the *TcCTL16* transcript level post-*E. coli* challenge. In addition, the expression of *rel*, a TF of the IMD signaling pathway, was upregulated in the *E. coli*-challenged group (Figure 7B). Furthermore, the results showed that the *E. coli*-induced expression of seven AMP genes (*att2*, *def1*, *def2*, *cole1*, *cole2*, *cecr2*, and *cecr3*) was upregulated, while the expression of *att1*, *att3*, and *def3* was not compared to the control beetles (ds-*GFP* + *E. coli*) (Figure 7C). Moreover, the survival rate of *TcCTL16*-RNAi *T. castaneum* infected with Gram-positive *S. aureus* (Figure 7D) and Gram-negative *E. coli* (Figure 7E) increased significantly compared with the control group. Taken together, our results suggest that loss of *TcCTL16* after RNAi could be compensated for by increasing AMP expression via the IMD pathways in *T. castaneum*.

## 3. Discussion

As a superfamily of proteins, CTLs bind to ligands with characteristic modules of one or more CRDs in a Ca^+^-dependent manner [31]. The functions of animal CTLs have been well studied in the past 2–3 decades [32,33,34], but information about the roles of CTLs in *T. castaneum* is relatively scarce. In this study, a CTL-X gene (*TcCTL16*) from *T. castaneum* was functionally characterized for the first time in insects. Our results show that TcCTL16 influences growth and development in early larvae, and more interestingly, TcCTL16 silencing can be compensated for by increasing AMP expression in late larvae in *T. castaneum*. 

TcCTL16, a CTL-X protein, contains one CRD domain, four CCPs, and one transmembrane domain. Interestingly, it does not contain a signal peptide but instead contains a transmembrane protein, suggesting that it may be secreted by a non-classical secretory pathway [35]. Additionally, the non-classical secretory pathway is closely related to cell proliferation, immune response, tumor formation and infectious disease pathology [36], suggesting that TcCTL16 has extensive functions in *T. castanuem*.

We found that *TcCTL16* was expressed at all stages of development and was especially highly expressed in the LP and EA stages. Pupation is an important stage of metamorphosis for holometabolous insects [28]. High expression of *TcCTL16* in late pupae suggests that *TcCTL16* might play an important role in the development and metamorphosis of *T. castaneum.* Our results showed that *TcCTL16* RNAi in early larvae shortened their body length and narrowed their body width, leading to the death of 98% of the larvae in the pupal stage. This phenomenon is consistent between the *uif* (CTL-X) of flies and the *TcCTL5* and *TcCTL12* of *T. castaneum*, the absence of which leads to death primarily in larvae [1,28,37]. Studies have shown that as a developmentally regulated protein, lectins are possibly involved in the recognition and fusion process during the myoblast fusion phase [38,39,40]. It is well known that muscle morphology and remodeling occur to accommodate the needs of insects during the metamorphosis of insects [41,42]. On this basis, we further examined the transcription of genes associated with muscle regulation and composition after *TcCTL16* knockdown. Compared with the control group, the transcript levels of muscle regulation genes, including *cut*, *vg*, *ewg*, *ap*, *salm*, and *Ims*, were obviously down-regulated, and muscle-composition-related genes (*Mhc* and *Mlc*) were also decreased. All of the above studies indicate that *TcCTL16* may be involved in the growth and development of *T. castaneum* by regulating muscle-related genes. 

In addition, our results showed that the mRNA of *TcCTL16* exhibited relatively high levels in the hemolymph, which was consistent with the distribution characteristics of C-type lectins in *T. castaneum* [1,27,28,43]. Similarly, some C-type lectins in silkworms (e.g., BmLBP, BmMBP, BmLEL-3, and BmCTL-S3) and mosquitoes (e.g., *AsCTLMA15*, *AsCTLGA5*, and *AsCTL15*) are mainly found in the hemolymph or hemocytes [8,44,45,46,47]. As an important immune component of invertebrates, hemolymph plays extremely important functions in defense by mediating nodule formation and encapsulation and by the exocytosis of a battery of bioactive molecules, such as AMPs [48]. Next, the transcript level of *TcCTL16* was obviously upregulated after injection with LPS, PGN, *E. coli*, and *S. aureus*. This expression pattern was consistent with the stimulation of CTLs in other invertebrates. For instance, the transcription of *Eriocheir sinensis EsCTL* [49], *M. sexta* immulectin-2 [50], *Portunus trituberculatus PtCLec2* [51], and *B. mori* C-type lectin 5 [52] were induced significantly after bacterial challenge. The results suggest that *TcCTL16* could be involved in the immune response to microbial infection in *T. castaneum*.

The function of most insect C-type lectins is to bind to bacteria by interacting with carbohydrates on the bacterial surface. Our study showed that rCTL16 displayed a Ca^2+^-dependent ability to bind to Gram^+^ bacteria (*S. aureus* and *B. thuringiensis*) and Gram^−^ bacteria (*E. coli* and *P. aeruginosa*) but had no binding activity on *B. subtilis*. Similar to the BmMBPs from *B. mori* [47], they have a high affinity to a wide range of Gram^+^ and Gram^-^ bacteria, although with different intensities. The lack of an ability to bind rCTL16 to *B. subtilis* suggests that it may not be able to recognize this bacterium. We know that the binding of C-type lectins to the surface of a pathogen may be the first step in their recognition and the initiation of defense mechanisms in innate immunity [12]. Therefore, the binding ability of rCTL16 towards microorganisms suggests its functions in recognizing these foreign intruders. Bacterial agglutination is an important biological role of C-type lectins. Our results further showed that rCTL16 was capable of agglutinating Gram^+^ bacteria (*S. aureus* and *B. thuringiensis*) and Gram^−^ bacteria (*E. coli* and *P. aeruginosa*) in a calcium-dependent manner, but it had no agglutinating ability against *B. subtilis*. Similar agglutination activity has also been observed in multi-CTLs from invertebrates, such as MsIML-2 from *M. sexta* [53] and LvPLP from *Litopenaeus vannamei* [54]. These results indicate that TcCTL16 has the ability to recognize *S. aureus*, *B. thuringiensis*, *E. coli*, and *P. aeruginosa* and agglutinate these bacteria in a calcium-dependent manner. 

Furthermore, the recognition and agglutination activity of CTLs is the initial stage of the defense mechanism. Most CTLs can induce a range of immune responses to clear the bacteria, fungi, and viruses, such as regulating the expression of AMPs [55]. *MjCC-CL* from *Marsupenaeus japonicus* can regulate the expression of AMPs by directly activating the JAK/STAT pathway to protect against bacterial infection [11]. Next, an RNAi experiment was performed to further prove the function of *TcCTL16* in regulating AMPs. Our results showed that the transcription of 7 AMPs (*att2*, *def1*, *def2*, *cole1*, *cole2*, *cecr2*, and *cecr3*) was obviously increased after *TcCTL16* knockdown in late larvae under *E. coli* stimulation, suggesting that loss of *TcCTL16* after RNAi could be compensated for by the increased expression of AMP genes. It has been reported that *T. castaneum* exhibit resistance to pathogen infection mainly through the expression of AMPs regulated by the Toll and IMD pathways [26]. The transcription factors of these two signaling pathways are *dif1* and *dif2* (Toll) and *rel* and *jnk* (IMD) [56]. By examining the expression pattern of transcription factors, we found that the transcript levels of *rel* were significantly upregulated after *TcCTL16* RNAi under *E. coli* infection, suggesting that *TcCTL16* loss could increase AMPs via the IMD pathway. Moreover, compared with the control group, the survival rate of *TcCTL16*-RNAi *T. castaneum* infected with *E. coli* and *S. aureus* increased significantly. These results directly supported the conclusion that the decreased mortality rate observed following bacterial injection of the ds-*TcCTL16*-treated beetles was primarily caused by enhanced larval resistibility to bacterial infection. In short, *T. castaneum* was able to compensate for the loss of *TcCTL16* by increasing AMP expression, thus increasing the survival rate in *TcCTL16* knockdown beetles.

In summary, we functionally characterized a CTL-X protein, TcCTL16. The results showed that *TcCTL16* was expressed at all stages of development, and *TcCTL16* silencing in early larvae influenced the growth and development of *T. castaneum*. Moreover, TcCTL16 could bind to microorganisms in a Ca^2+^-dependent manner, and the loss of *TcCTL16* in late larvae could be compensated for by the increased expression of AMP genes via the IMD immune pathway, thus decreased mortality in *TcCTL16* knockdown beetles. Together, our sequence comparison and function analysis of TcCTL16 establish a solid foundation for future studies of *T. castaneum* CTL proteins. 

## 4. Materials and Methods

### 4.1. Animals and Chemicals

The *T. castaneum* Georgia-1 (GA-1) strain was used for all experiments in this study. Beetles were reared in whole wheat flour containing brewer’s yeast (5%) at 30 °C, 40% relative humidity, and a 14:10 h light:dark photoperiod [27]. LPS from *E. coli* and PGN from *S. aureus* were obtained from Sigma (St Louis, MO, USA). The pET-28a (+) vectors *E. coli* Trans1T1 and *E. coli* BL21 (DE3) were purchased from TransGene (Beijing, China). A one-step cloning kit was purchased from Vazyme (Nanjing, China).

### 4.2. Identification and Cloning of TcCTL16 Gene

A pool of three 15-day-old larvae was used to isolate total RNA, and then 1 μg total RNA was converted to cDNA using a previously described method [43,57]. The full-length cDNA sequence of *TcCTL16* was downloaded from Beetlebase (http://www.beetlebase.com/ accessed on 22 June 2023). *TcCTL16* was amplified by RT-PCR with the primers *TcCTL16*-F and *TcCTL16*-R (Table 1) and sequenced for confirmation by Springen Biotechnology Company (Nanjing, China). A similarity analysis was conducted using BLAST (http://www.ncbi.nlm.nih.gov/ accessed on 22 June 2023). The corresponding cDNA was conceptually translated, and the deduced proteins were predicted using ExPASy (http://www.expasy.org/ accessed on 22 June 2023). 

### 4.3. Sequence and Phylogenetic Analysis

The CTL amino acid sequences of *T. castaneum* were obtained from Beetlebase (http://www.beetlebase.org/ accessed on 22 June 2023) and the National Center for Biotechnology Information (NCBI) database (https://www.ncbi.nlm.nih.gov/ accessed on 22 June 2023). The CTL amino acid sequences of *Habropoda laboriosa* and *Anoplophora glabripennis* were obtained from the NCBI database (https://www.ncbi.nlm.nih.gov/ accessed on 22 June 2023). The CTL sequences of *Manduca sexta* and *Bombyx mori* were obtained from the literature [5,18]. The CTL sequences of *Anopheles gambiae* and *D. melanogaster* were obtained from http://cegg.unige.ch/Insecta/immunodb accessed on 22 June 2023. These sequences were aligned with ClustalW2 (http://www.ebi.ac.uk/Tools/msa/clustalw2/ accessed on 22 June 2023). The neighbor-joining trees were constructed with 1000 bootstrap replicates in MEGA7. Domain architecture prediction of the proteins was performed using SMART (http://smart.embl-heidelberg.de/ accessed on 22 June 2023). The SignalP 4.1 program was used to predict the presence and location of the signal peptide (http://www.cbs.dtu.dk/services/SignalP4.1/ accessed on 22 June 2023). The potential disulfide bonds and their positions were predicted using the Scanprosite program (http://www.expasy.ch/tools/scanprosite/ accessed on 22 June 2023).

### 4.4. Spatiotemporal Expression of TcCTL16

For the stage-specific expression study, total RNA was extracted from pools of multiple individuals in the following developmental stages: early eggs (EE, 1 day old, ~50 mg), late eggs (LE, 3 days old, ~50 mg), early larvae (EL, 1 day old, ~50 mg), late larvae (LL, 20 days old, 3 individuals), early pupae (EP, 1 day old, 3 individuals), late pupae (LP, 5 days old, 3 individuals), early adults (EA, 1 day old, 3 individuals), and late adults (LA, 10 days old, 3 individuals). For the tissue-specific expression study, total RNA was also extracted from pools of approximately 100 15-day-old larval tissues, including gut, fat body, epidermis, hemocytes, and Malpighian tubule tissues. Reverse transcription was performed using 1 μg total RNA. Quantitative real-time PCR (qRT-PCR) was employed to analyze the expression patterns of *TcCTL16* with a SYBR Green Master Mix (Vazyme, Nanjing, China) following the manufacturer’s instructions by the StepOnePlus Real-Time PCR System (ABI). The primer sequences (*TcCTL16*-qF and *TcCTL16*-qR) for *TcCTL16* are listed in Table 1. *T. castaneum* ribosomal protein S3 (*Tcrps3*) was used as the internal reference [58]. The relative expression levels of *TcCTL16* were calculated according to the 2^−ΔΔCT^ method. The experiments were carried out in three technique repeats and three biological replicates.

### 4.5. Expression Profiles of TcCTL16 after Being Challenged with Polysaccharides and Bacteria

To measure *TcCTL16* expression after LPS, PGN, *S. aureus*, and *E. coli* challenges, 12-day-old larvae were collected and separated into three groups. Approximately 60 synchronous larvae in each group were injected with LPS, PGN, *S. aureus*, *E. coli*, or physiological buffer (0.373 g/L KCl, 0.038 g/L Na_3_PO_4_·12H_2_O, pH 7.0). Pools of three randomly selected larvae were sampled for RNA isolation at 12, 24, 36, 48, 60, and 72 h post-injection to reveal the temporal expression profile of *TcCTL16. TcCTL16* expression levels in the beetles challenged with polysaccharides and bacterium were determined via qRT-PCR as described in 2.4. The primer sequences of *TcCTL16* (*TcCTL16*-qF and *TcCTL16*-qR) are listed in Table 1. Three technical repeats for each biological replicate and a total of three biological replicates were performed.

### 4.6. Production of Recombinant TcCTL16 and Western Blot Analysis

The partial cDNA fragment encoding the CRD domain of TcCTL16 was amplified with primers (TcCTL16-F and TcCTL16-R, Table 1). The fragments were then ligated into pET-28a (+) plasmids with His-tag using the Hieff Clone Plus One Step Cloning Kit (Yeasen, Shanghai, China) following the manufacturer’s instructions, which were further transformed into *E. coli* BL21 (DE3) cells for protein expression. The recombinant CRD protein (designated rCTL16) was expressed in insoluble form and purified by a Ni Sepharose 6 Fast Flow column (GE Health care, Marlborough, MA, USA) under denatured conditions (8 M urea). The purified protein was refolded in gradient urea-TBS glycerol buffer according to Wang et al. [59]. Then, the resultant proteins were separated by 12.5% reducing sodium dodecyl sulfate polyacrylamide gel electrophoresis (SDS-PAGE) and visualized with Coomassie brilliant blue R-250 (Jiancheng, Nanjing, China). At the same time, the resultant proteins were transferred to polyvinylidene difluoride (PVDF) membranes using Bio-Rad Criterion (Mini Trans-Blot, Bio-Rad, Hercules, USA), and then the membranes were blocked in 10 mL of TBS containing 5% bovine serum albumin (BSA, 771407, AiKB, Qingdao, China) for 2 h at room temperature (RT). The membranes were washed three times with TBS containing 0.05% Tween-20 (TBST) and incubated overnight at 4 ℃ with anti-His rabbit polyclonal antibodies (Transgen, Beijing, China) at a 1: 2000 dilution in 5% BSA. After incubation, the membranes were washed four times with TBST and incubated at RT for 2 h with a goat anti-rabbit IgG horseradish peroxidase (HRP) conjugate (Transgen, Beijing, China) diluted 1:5000 in TBST. The bands were detected by incubation with an enhanced chemiluminescence (ECL) substrate solution (E411-01/02) according to the manufacturer’s instructions (Vazyme, Nanjing, China).

### 4.7. Microorganism Binding and Agglutination of TcCTL16

Five types of microorganisms, including three Gram^+^ bacteria (*S. aureus*, *B. subtilis*, and *Bacillus thuringiensis*) and two Gram^−^ bacteria (*E. coli*, *Pseudomonas aeruginosa*), were used to test the binding spectrum of rCTL16 as previously described [27]. The bacteria were cultured overnight and centrifuged at 6000× *g* for 5 min at RT. The resulting bacterial pellets were washed three times with TBS and resuspended in TBS at 2 × 10^8^ cells/mL. In the presence of 10 mM Ca^2+^, purified rCTL16 were incubated respectively with each of the five microorganisms mentioned above for 1 h at RT with gentle rotation. The microorganisms were pelleted and washed four times with 1 mL of TBS. The pellet was resuspended in 100 µL of 7% SDS solution and centrifuged at 6000× *g* for five min. The supernatant was collected for Western blotting as described in Section 2.6.

Five types of microorganisms, as mentioned above, were also used to detect the direct agglutination ability of rCTL16 according to a previously described method [1]. The equal volume of bacterial suspension (5 × 10^6^ bacterial count in 25 µL) was incubated with rCTL16 (100 µg/mL) at 28 °C for 1 h in the presence of 10 mM Ca^2+^. The recombinant methyl-CpG-binding domain (rTcMBD) with a His tag from *T. castaneum* was used as a negative control. Agglutination reactions were observed using a compound light microscope with a 1000× objective.

### 4.8. dsRNA-Mediated RNAi Assay

The double-stranded RNA (dsRNA) targeting the *TcCTL16* was synthesized with an in vitro transcription T7 kit for dsRNA synthesis (Takara, Kyoto, Japan). The DNA template for the ds-*TcCTL16* preparation was generated by RT-PCR using the gene-specific primers ds-*TcCTL16*-F and ds-*TcCTL16*-R (Table 1). Afterward, the formation of dsRNA was monitored by determining the molecular size using agarose gel electrophoresis and quantified by spectrophotometry. The length of the ds-*TcCTL16* was 530 bp. The dsRNA targeting *GFP* (as control) was synthesized as previously described [27].

To investigate the RNAi’s efficiency, a total of 200 ng ds-*TcCTL16* in 200 nL was injected into the body cavity of each 12-day larva. Beetles injected with an equal volume of ds-*GFP* were set as control groups. Total RNA was isolated from pools of three larvae of ds-*TcCTL16* and ds-*GFP* groups on days 1, 2, and 3 after injection. Then, a total of 1 μg RNA was converted to cDNA, and qRT-PCR was performed to quantify the *TcCTL16* level using the method described in Section 2.4. 

The 12-day-old larvae were injected with the ds-*TcCTL16* to test its influence on the growth and development of *T. castaneum*. Transcript levels of genes involved in muscle regulation and composition were measured with the primers listed in a published article [1].

To further explore whether *TcCTL16* participates in the immune response through regulating the expression of AMP and transcription factor (TF) genes in red flour beetles, we injected ds-*TcCTL16* into 15-day-old larvae and then challenged them with *E. coli* after 3 days using the above method. When *TcCTL16* was knocked down by RNAi, transcript levels of ten AMPs and four TFs were measured at 60 h post bacterial challenge with qRT-PCR using the primers listed in a published paper [1,27]. Three technical repeats per replicate and three biological replicates were carried out for each experiment.

### 4.9. Survival Assay

*T. castaneum* were divided into four groups (30 larvae per group) to evaluate the larva survival rate for *S. aureus* and *E. coli* infection after *TcCTL16* knockdown. ds-*GFP* was used as a control. After *TcCTL16* was knocked down by ds-TcCTL16 injection, all larvae were injected with 200 nL of *S. aureus* (about 7.0 × 10^4^ cells) or *E. coli* (about 7.8 × 10^4^ cells) at 72 h post-dsRNA injection. The number of dead larvae was monitored every 12 h, and the survival rates were calculated. The experiments were repeated three times. 

### 4.10. Statistical Analyses

The gene expression data and the mean values of the RNAi-treated larvae versus the mean values of the control larvae were compared by one-way analysis of variance (ANOVA) in combination with Student’s *t*-test using the SPSS version 13.0 statistics program (SPSS Inc., Chicago, IL, USA). All data obtained were presented as the mean ± SD from three independent experiments. Asterisks indicate statistical significance (* *p* < 0.05, ** *p* < 0.01, and *** *p* < 0.001).

## Figures and Tables

**Table 1:** Oligonucleotide primers used in the current study.

Name	Sequence (5′-3′)
PCR:	
*TcCTL16*-F	ATGGTCGTGTGTCAGTGCAAGG
*TcCTL16*-R	ATATCTCTTCTGGTCGCGGTGC
TcCTL16-F	atgggtcgcggatccgaattcATGGTCGTGTGTCAGTGCAAGG
TcCTL16-R	gtggtggtggtggtgctcgagATATCTCTTCTGGTCGCGGTGC
RNAi:	
ds-*TcCTL16*-F	taatacgactcactatagggATGGTCGTGTGTCAGTGCAA
ds-*TcCTL16*-R	taatacgactcactatagggATTGTGGCAGGTTTGTTCGC
qRT-PCR:	
*TcCTL16*-qF	TGTGAACCTGTCCAGTGCG
*TcCTL16*-qR	CATCATACTGTCCATTCGCC

## Data Availability

The datasets generated and/or analyzed in this study are available from the corresponding author upon reasonable request.

## Supplementary Materials

The following supporting information can be downloaded at: https://www.mdpi.com/article/10.3390/ijms241310700/s1.
