# Supplementary figures and images for: Functional Analysis of a CTL-X-Type Lectin CTL16 in Development and Innate Immunity of Tribolium castaneum
Source: Int J Mol Sci. 2023 Jun 27;24(13):10700. doi: 10.3390/ijms241310700 (PMC10341621; doi:10.3390/ijms241310700)

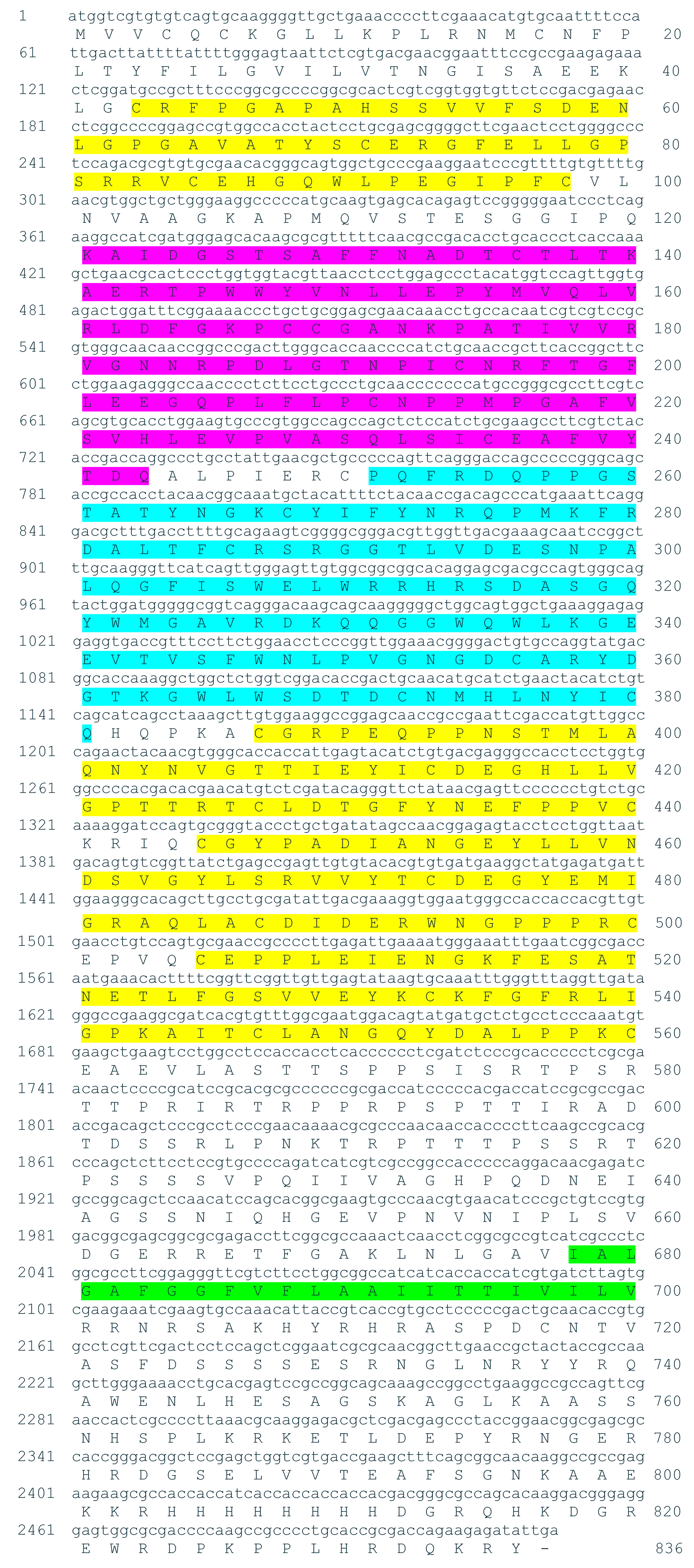

Supplement: Supplementary file 1 [file ijms-24-10700-s001.zip › Fig. S1.jpg]

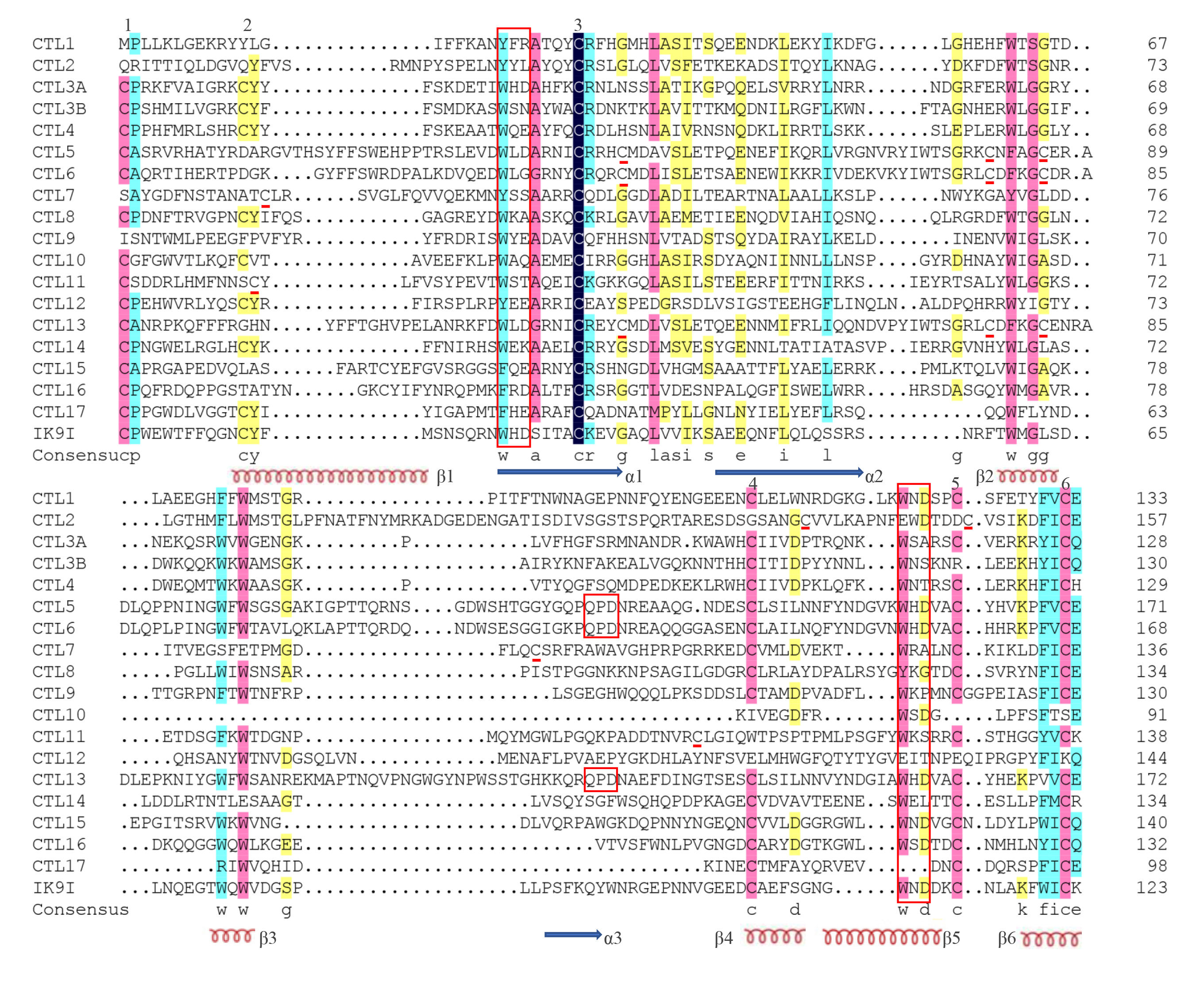

Supplement: Supplementary file 1 [file ijms-24-10700-s001.zip › Fig. S2.jpg]

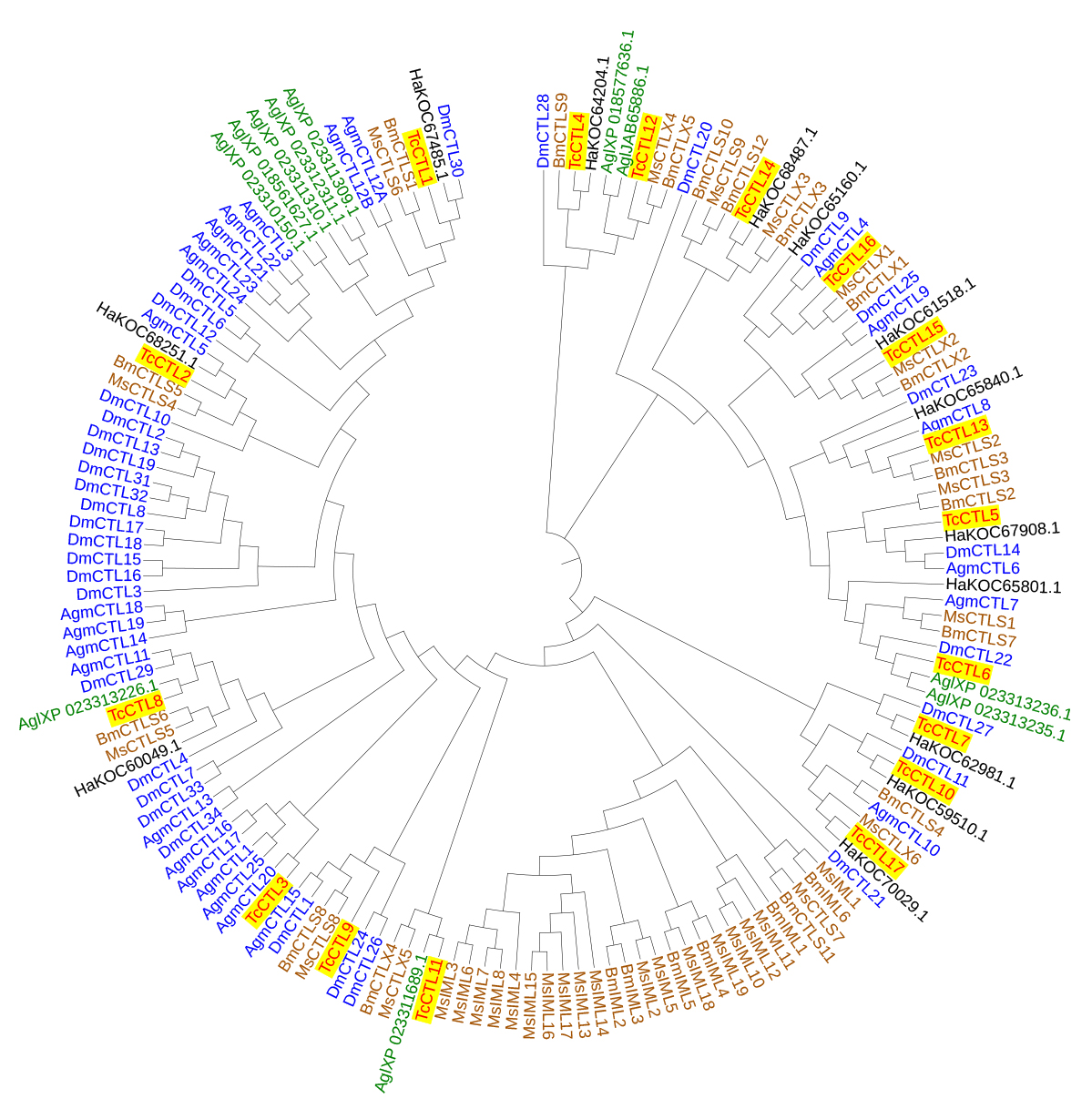

Supplement: Supplementary file 1 [file ijms-24-10700-s001.zip › Fig. S3.jpg]
